# The Phylogeny of Brassicaceae YABBYs and the *CRC*-Mediated Regulation of Stigma Development in *Brassica napus*

**DOI:** 10.3390/ijms27135740

**Published:** 2026-06-25

**Authors:** Lin Dai, Jinxiang Gao, Cheng Li, Tao Han, Zhengshu Tian, Yunyun Zhang, Yusong Zhang, Yanqing Luo, Kaiqin Zhao, Xiaoyan Yuan, Canzhi Zhang, Tao Liu, Feng Zu, Pei Qin

**Affiliations:** 1 College of Agronomy and Biotechnology, Yunnan Agricultural University, Kunming 650201, China; 2024240239@stu.ynau.edu.cn (L.D.); 2025240281@stu.ynau.edu.cn (C.L.); bohuyeshan@163.com (T.H.); yantao618@126.com (T.L.); 2 Yunnan Key Laboratory of Genetic Improvement of Herbal Oil Crops, Industrial Crops Research Institute, Yunnan Academy of Agricultural Sciences, Kunming 650225, China; gaojx@yaas.org.cn (J.G.); zs_tian2017@163.com (Z.T.); yunyunsky111@163.com (Y.Z.); zhangys@yaas.org.cn (Y.Z.); luoyq@yaas.org.cn (Y.L.); zkq@yaas.org.cn (K.Z.); yuanxiaoyan69604@163.com (X.Y.); 13368808407@163.com (C.Z.)

**Keywords:** YABBY gene family, *Brassicaceae* species, *Brassica napus*, *CRC*, programmed cell death

## Abstract

The YABBY family consists of plant-specific transcription factors that regulate organ polarity and reproductive development. As a member of this family, *CRABS CLAW* (*CRC*) plays crucial roles, but its molecular mechanism in oilseed rape stigma development remains unclear. In this study, we identified YABBY genes in four *Brassicaceae* species. The results showed that CRC proteins are highly conserved in structure, but their cis-acting elements vary among species. To explore its function, we performed transcriptome sequencing on an oilseed rape *CRC*-deficient mutant (*sd*). The transcriptome data revealed multiple changes in the *sd* mutant. Specifically, brassinosteroid (BR) signaling factors were downregulated. Sugar transporters and auxin-related genes showed abnormal expression. Furthermore, pro-senescence and programmed cell death (PCD) genes were upregulated, whereas the classic senescence pathway remained unchanged. Based on these findings, we propose a potential mechanism. The loss of *CRC* disrupts BR signaling, sugar transport, and calcium homeostasis. This disruption triggers non-classic death of stigma papilla cells, which hinders pollen tube penetration and reduces seed set. Notably, increasing environmental humidity partially rescued the seed set, likely by delaying cell death. Although these transcriptomic insights warrant further experimental validation, this study provides valuable clues and genetic resources for future research on reproductive development in oilseed rape.

## 1. Introduction

The family Brassicaceae comprises numerous economically important crops. Among these, rapeseed (*Brassica napus*) is one of the most significant oilseed crops in China, with its yield heavily dependent on normal floral organ development. The YABBY family is a plant-specific transcription factor family characterized by two highly conserved domains: an N-terminal C2C2 zinc finger domain and a C-terminal YABBY domain [1,2,3,4,5,6,7,8]. This family originated from the ancestors of seed plants and can be found in both gymnosperms and angiosperms. Depending on the study, the YABBY family is divided into four [9], five [10,11,12,13,14], or six subfamilies [2,15]. Based on the expression patterns and functional preferences, YABBY genes can be further categorized into two groups, vegetative and reproductive. The former includes members such as *FIL/YAB1*, *YAB2*, *YAB3*, and *YAB5*, which mainly regulate vegetative organ development; the latter is represented by *CRABS CLAW* (*CRC*) and *INNER NO OUTER* (*INO*), which specifically participate in the formation of reproductive organs such as pistils and ovules, playing key roles in reproductive development [16]. In Brassicaceae plants, the YABBY gene family has expanded due to lineage-specific whole-genome triploidisation events, with different subfamilies exhibiting distinct patterns of gene retention and loss [17,18]. Comparative genomics analyses of species in the Brassicaceae family, such as *Arabidopsis thaliana*, *Brassica rapa*, *Brassica oleracea* and *Brassica napus* (cabbage-type rapeseed), indicate that the *FIL/YAB3* subfamily has undergone significant, dose-dependent expansion within the genus *Brassica*. In contrast, the *CRC* subfamily has consistently remained in a single- or low-copy state throughout evolution, suggesting that its function is subject to strong purifying selection [17,18].

*CRC* is one of the most evolutionarily conserved members of the YABBY family, playing a central regulatory role in the reproductive development of angiosperms [19,20,21]. It is a key regulator of carpel development and also plays an important role in nectary development and floral meristem termination. The function of *CRC* in carpel development is highly conserved across diverse plant species, including *Arabidopsis* [1,22,23,24,25], rice [26], tomato [27], Physalis [28], Asparagus [29], and cucumber [3,30]. Functional studies have demonstrated that *CRC* is involved in floral meristem termination in *Arabidopsis*, rice, maize, and tomato [5,31,32,33]; it participates in nectary development in core eudicots, whereas such expression features have not been observed in basal angiosperms or most monocots [22]. Furthermore, *CRC* functions diverge among different lineages: it regulates vascular bundle development in pea, participates in embryogenesis in California poppy, affects leaf midrib formation in monocots, and is more associated with nectary development in dicots [26,29].

*Brassica napus* (*B. napus*, AACC, 2n = 38) is an allogamous tetraploid species formed by chromosome doubling following a natural hybrid between *Brassica rapa* (*B. rapa*, AA, 2n = 20) and *Brassica oleracea* (*B. oleracea*, CC, 2n = 18). Previous studies conducted genome-wide identification of the YABBY gene family in cabbage-type rapeseed, identifying a total of 79 YABBY genes; phylogenetic analysis revealed their evolutionary relationships within the genus Brassica [17]. In a previous study, we used a map-based cloning strategy to localize the stigma development defect mutant (*sd*) in *B. napus* rapeseed. Its target genes were *BnaCRCs* (*BnaA07.CRC* and *BnaC06.CRC*), which correspond to *BnYAB6.1* and *BnYAB6.2*, respectively. This mutant exhibits phenotypes such as the collapse of stigma papillae, the absence of nectaries, defects in pollen tube guidance, significantly reduced female fertility, and vacuolar rupture in papillae 16 h after self-pollination [34,35], directly confirming the essential role of *CRC* in stigma development in the genus *Brassica*.

To systematically elucidate the biological function of *CRC* genes in rapeseed, this study first performed a comprehensive phylogenetic analysis of the YABBY gene family in four representative Brassicaceae species (*B. napus*, *B. rapa*, *B. oleracea*, and *A. thaliana*) to clarify the evolutionary position of *CRC* and its functional conservation and specificity. Secondly, we conducted comparative transcriptome analysis of stigma tissues from 2 to 4 mm flower buds of the *sd* mutant and wild-type plants. This analysis focused on differentially expressed genes involved in stigma papilla cell morphogenesis, stigma secretory function, cell wall remodeling required for pollen tube penetration, pollen tube recognition and entry signals, self-incompatibility and defense rejection, cellular senescence, and programmed cell death (PCD). This study aims to provide effective clues for unraveling the molecular mechanism by which *CRC* genes regulate stigma development and seed set in rapeseed. Our work not only offers new insights into the evolutionary patterns of the YABBY family and functional divergence of *CRC* genes in rapeseed. It also reveals the core regulatory role of *CRC* in rapeseed reproductive development, thereby providing important theoretical basis and genetic resources for the genetic improvement of yield traits in rapeseed.

## 2. Results

### 2.1. Identification and Naming of the YABBY Gene Family in Brassicaceae

In this study, YABBY gene family members were systematically identified in four representative Brassicaceae species, namely *Brassica napus* (*B. napus*), *Brassica rapa* (*B. rapa*), *Brassica oleracea* (*B. oleracea*), and *Arabidopsis thaliana* (*A. thaliana*), while *B. rapa* (AA, 2n = 20) and *B. oleracea* (CC, 2n = 18) are known to be *B. napus* (AACC, 2n = 38) progenitor species. The results showed that 22, 11, 11, and 6 YABBY genes were identified in the four species, respectively, with their C2C2 zinc finger domains and YABBY domains presented in Figure S1. To facilitate subsequent research and communication, the YABBY genes from these four species were uniformly named based on their physical positions on chromosomes and the results of homology analysis with known *Arabidopsis* YABBY genes (Table 1). In *B. napus*, 22 YABBY genes were identified, belonging to six subfamilies (*YAB1*, *YAB2*, *YAB3*, *YAB4*/*INO*, *YAB5*, *YAB6*/*CRC*). Each subfamily was named after the corresponding *Arabidopsis* subfamily, i.e., *BnYAB1* to *BnYAB6*. Different copies within the same subfamily were sequentially numbered according to their physical positions on chromosomes; for example, the six copies of the *YAB1* subfamily were named *BnYAB1.1*–*BnYAB1.6* (see Table 1 for details). The YABBY genes in *B. rapa* and *B. oleracea* were named *BrYAB1*–*BrYAB6* and *BoYAB1*–*BoYAB6*, respectively, following the same naming rules for homologous copies as in *B. napus*. The YABBY genes in *A. thaliana* retained the previously reported standard names (e.g., *AtINO*, *AtCRC*).

### 2.2. Bioinformatics Analysis of the YABBY Gene Family in Brassicaceae

The molecular weights (MW) of *Brassicaceae* YABBY proteins ranged from 13.0 to 33.1 kDa, with amino acid lengths varying from 117 to 294, indicating certain structural differences among members, though all belong to small- to medium-sized proteins. Specifically, for *B. napus* YABBY proteins (BnYABs),the MW range from 13.0 to 26.5 kDa (with an average of 22.9 kDa),and the amino acid length ranged from 117 to 238 aas (with an average of 205); for *B. rapa* (BrYABs) and *B. oleracea* (BoYABs) YABBY proteins, the MWs ranged from 18.3 to 32.7 kDa (with an average of 24.4 kDa) and from 18.2 to 33.1 kDa (with an average of 25.0 kDa), corresponding to amino acid lengths from 164 to 286 aas (with an average of 217) and from 164 to 294 aas (with an average of 228), respectively; for *Arabidopsis* YABBY proteins (AtYABs), the MW range from 18.5 to 29.6 kDa (with an average of 23.4 kDa), and the amino acid length ranged from 164 to 262 aas (with an average of 210) (Figure S2). The isoelectric points (pI) of these proteins ranged from 5.39 to 9.75, with average pI values of 8.12, 8.50, 8.56, and 8.38 for BnYABs, BrYABs, BoYABs, and AtYABs, respectively (Figure S2). Most of these proteins are basic, suggesting they are rich in positively charged amino acid residues, which facilitates binding to negatively charged DNA molecules—a property consistent with their role as transcription factors involved in gene expression regulation. Notably, as indicated in Figure S2, the average amino acid lengths, molecular weights, and pI values of YABBY proteins were highly similar across all analyzed species. Subcellular localization predictions indicated that all 50 YABBY proteins are localized in the nucleus (Table 1 and Table S2), which aligns with the functional characteristics of YABBY transcription factors.

### 2.3. Phylogenetic Analysis

**Figure 1:**
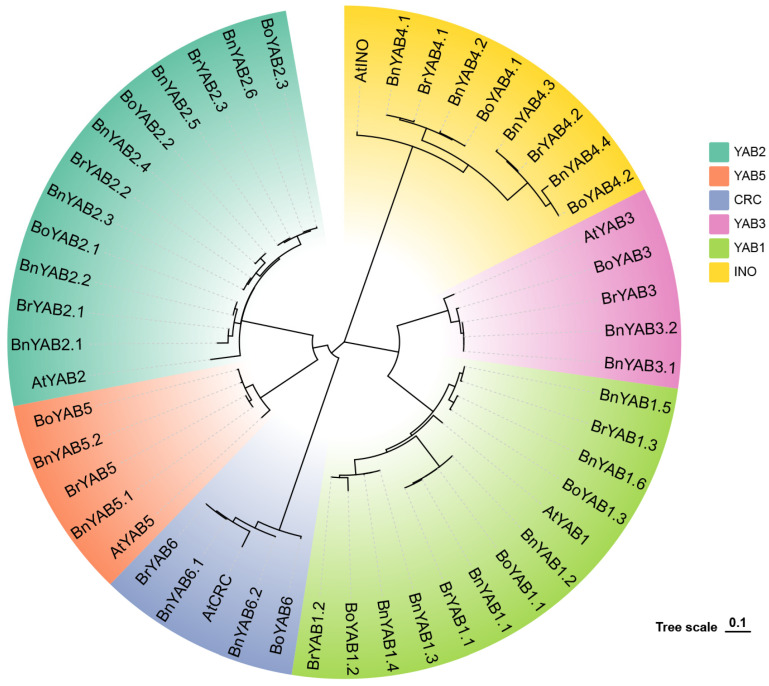
Phylogenetic analysis of the YABBY gene family in four representative Brassicaceae species.

To investigate the evolutionary relationships of YABBY gene family in four representative Brassicaceae species, a phylogenetic analysis was constructed using 50 YABBY proteins. The results showed that the YABBY gene family in Brassicaceae could be classified into six subfamilies (YAB1, YAB2, YAB3, INO, YAB5, and CRC) (Figure 1), which is consistent with previous studies [2,15]. Among these, the YAB1 and YAB2 subfamilies contained the largest number of members, each comprising 13 members: six in *Brassica napus*, three in *B. rapa*, three in *B. oleracea*, and one in *Arabidopsis thalian*. The INO subfamily had nine members (4, 2, 2, 1, respectively). The YAB3, YAB5, and CRC subfamilies each contained five members (2, 1, 1, 1, respectively). In each species, the CRC subfamily existed as a single orthologous gene rather than arising from ancestral duplication, which is in agreement with previous reports [10,22]. Both INO and CRC formed independent clades in the phylogenetic tree, indicating their distant relationships with the other subfamilies. This finding supports the view that these two subfamilies likely diverged early and assumed specialized functions [10].

### 2.4. Synteny and Chromosomal Localization Analysis

Synteny analysis revealed a substantial number of syntenic YABBY gene pairs among the four species (Figure S3). A one-to-multiple syntenic relationship was observed between *A. thaliana* and both *B. rapa* and *B. oleracea*, suggesting that the YABBY gene family underwent expansion during this period. The number of YABBY genes in the A and C subgenomes of *B. napus* (22 in total) was largely consistent with that in its progenitor species, *B. rapa* (11) and *B. oleracea* (11).

**Figure 2:**
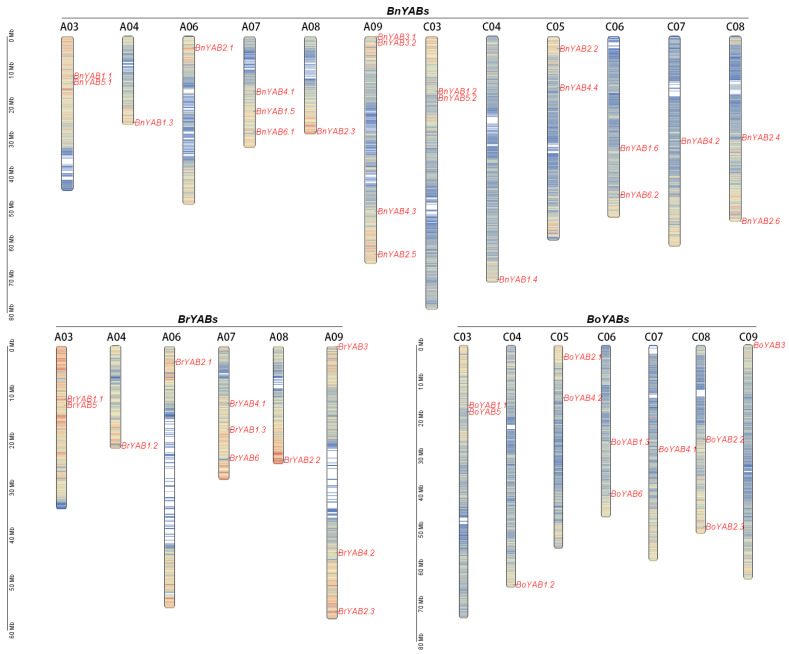
Chromosomal distribution of YABBY genes in three representative Brassicaceae species.

Chromosomal localization analysis showed that YABBY genes were unevenly distributed across the chromosomes of all four species (Figure 2; Table 1). In *B. rapa* and *B. oleracea*, 11 *BrYABs* and 11 *BoYABs* were identified, respectively. In *B. napus*, 22 *BnYABs* were located on 12 chromosomes of the A and C subgenomes. Among these, 12 genes were located on the A subgenome (A01–A10), predominantly on chromosomes A09 (4 genes) and A07 (3 genes); while ten genes were located on the C subgenome (C01–C09), with a more dispersed distribution. Comparative analysis indicated that the distribution and approximate chromosomal positions of YABBY genes on the A subgenome of *B. napus* were largely consistent with those on the genome of *B. rapa*. Similarly, the YABBY genes on the C subgenome of *B. napus* showed a high degree of consistency with those on the genome of *B. oleracea*. Only one exception was noted: the two *YAB3* copies on chromosome A09 of *B. napus* (*BnYAB3.1* and *BnYAB3.2*) corresponded to *BrYAB3* on chromosome A09 of *B. rapa* and *BoYAB3* on chromosome C09 of *B. oleracea*, respectively.

These results indicate that YABBY genes are highly conserved during the evolution of Brassicaceae species. Furthermore, as an allotetraploid, *B. napus* likely inherited part of the YABBY gene characteristics from *B. rapa* and *B. oleracea*, and to some extent, functional divergence may have occurred in these genes during this process.

### 2.5. Gene Structure and Conserved Domain Analysis

**Figure 3:**
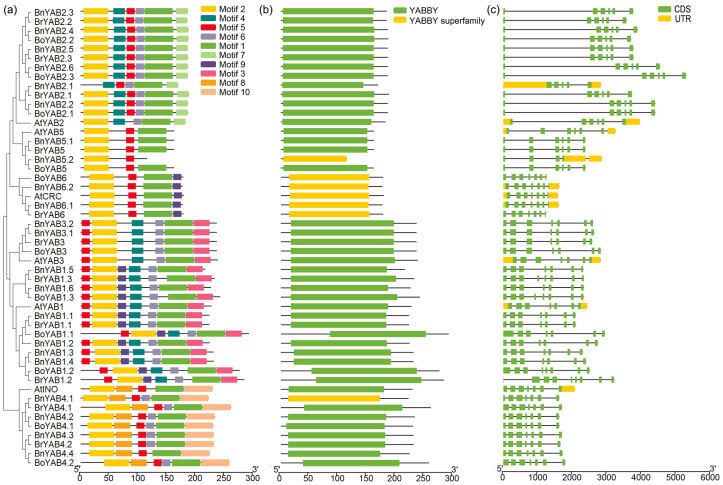
Motifs, conserved domains, and gene structures of YABBY genes in four representative Brassicaceae species. (**a**) Motif analysis: Distribution of conserved motifs (numbered 1–10). (**b**) Conserved domains: Distribution of diagnostic domains. (**c**) Gene structures: Exon–intron organization.

The gene structures of YABBY genes varied to some extent: most genes contained 6–7 exons, while a few had 4, 5, or 8 exons. Gene structures within the same subfamily exhibited high similarity, with the CRC and INO subfamilies showing particularly consistent exon numbers and distribution patterns, further validating their evolutionary conservation (Figure 3c). Motif analysis revealed that all YABBY proteins contained two core conserved motifs (motif 2 and motif 1) (Figure 3a), corresponding to the C2C2 zinc finger domain and the YABBY domain, respectively (Figure S4). The composition of conserved motifs was highly similar within each subfamily, indicating that these motifs are critical for YABBY protein function. Conserved domain prediction further confirmed that all YABBY proteins possess the typical C2C2 zinc finger and YABBY domains. Notably, the YABBY domain of CRC subfamily members showed the highest matching score with the YABBY superfamily (PF04690) in the Pfam database (Figure 3b), suggesting that this subfamily may have undergone stronger purifying selection.

### 2.6. Cis-Acting Element Analysis

The promoter regions of YABBY genes contained multiple cis-acting elements, which could be categorized into three major classes (Figure S5; Table S3): elements related to plant growth and development (e.g., meristem expression elements, endosperm expression elements, light-responsive elements, tissue-specific expression elements), hormone-responsive elements (e.g., auxin, ABA, GA, MeJA, SA), and abiotic stress-responsive elements (e.g., low-temperature response, wound response, drought-induced elements). Among these, the promoter regions of *CRC* subfamily genes were enriched in hormone-responsive elements and meristem expression elements, indicating that *CRC* genes may be regulated by multiple hormones and participate in the regulation of reproductive organ development. Notably, a number of orthologous gene pairs (e.g., *BnYAB4.4/BoYAB4.2*, *BnYAB4.3/BrYAB4.2*, and *BnYAB2.2/BoYAB2.1*) exhibited promoters that were almost identical in both the composition and spatial location of cis-acting elements, suggesting highly conserved regulatory mechanisms.

### 2.7. Expression Patterns of YABBY Genes in B. napus and Functional Analysis of the CRC Subfamily

**Figure 4:**
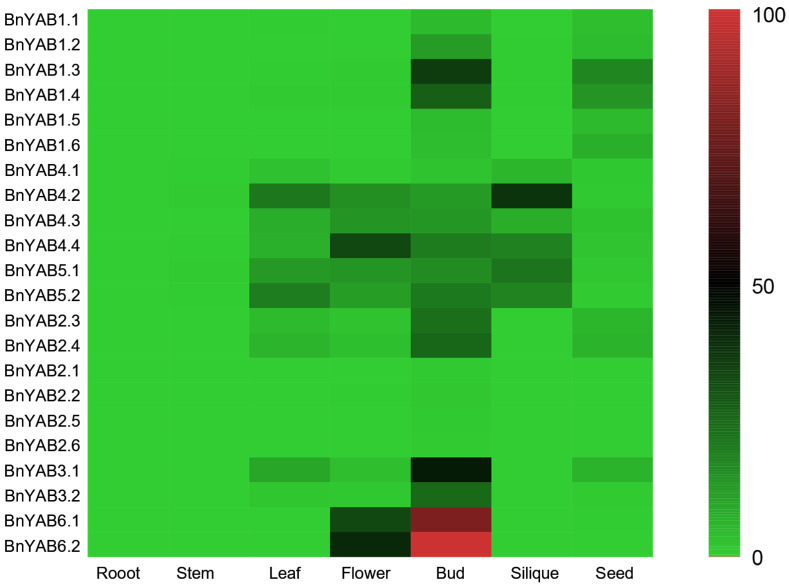
Expression patterns of 22 BnYAB genes in *B. napus*.

To investigate the expression patterns of BnYABs in different tissues, the transcript levels of 22 BnYAB genes were analyzed using public transcriptome data of *B. napus* (Figure 4). The results showed that BnYABs were barely expressed in roots and stems but were predominantly expressed in floral organs. Among them, *BnYAB6.1* and *BnYAB6.2*, which belong to the CRC subfamily, exhibited the highest expression levels in flower buds, suggesting that this subfamily may play an important role in floral organ development in *B. napus*.

Based on the systematic analysis of the YABBY gene family in four Brassicaceae species described above, this family is highly conserved evolutionarily, yet distinct functional differentiation has emerged among subfamilies. The CRC subfamily possesses three prominent characteristics: (i) it has undergone relatively strong purifying selection during evolution; (ii) its promoter regions are significantly enriched in hormone-responsive elements and meristem expression elements; and (iii) it shows high expression in buds. These results strongly suggest that CRC subfamily may have acquired specialized functions in reproductive organ (especially carpel and stigma) development. However, the precise molecular mechanisms of *CRC* genes in *B. napus* remain unclear.

**Figure 5:**
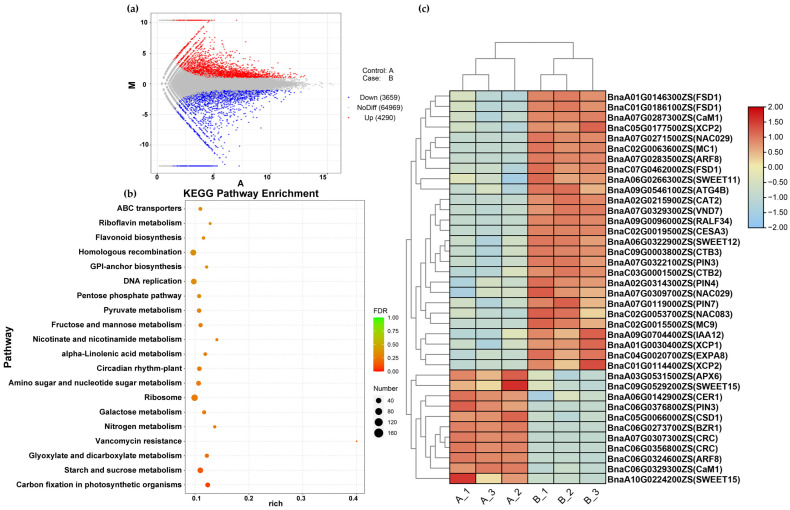
Transcriptome analysis wild-type and *sd* mutant. (**a**) Transcriptome sequencing of stigmas from 2 to 4 mm flower buds of wild-type and *sd* mutant plants; (**b**) KEGG pathway enrichment analysis of differentially expressed genes; (**c**) selected significantly differentially expressed genes in wild-type and *sd* mutant of *B. napus*.

To elucidate the function of *CRC* genes, we previously identified a stigma-defective mutant *sd* (stigma defective) in *B. napus*, in which the expression levels of two *CRC* homologous copies (*BnYAB6.1* and *BnYAB6.2*) were significantly downregulated. This mutant exhibited deflated and collapsed stigma papilla cells [34]; mature pollen grains grew randomly on the stigma surface and failed to penetrate the papilla cells, and vacuole rupture in papilla cells occurred 15 h after natural pollination [35]. To investigate the underlying molecular mechanism, we collected stigmas from 2 to 4 mm flower buds of wild-type and *sd* mutant plants and performed transcriptome sequencing analysis. The results revealed 4290 upregulated genes and 3659 downregulated genes in the *sd* mutant (Figure 5a). GO enrichment analysis indicated that the differentially expressed genes were significantly enriched in terms such as transcription coregulator activity, organic substance transport, carbohydrate transport, and anion transmembrane transport (Table S8). KEGG enrichment analysis showed that the differentially expressed genes were mainly enriched in pathways including carbon fixation in photosynthetic organisms, starch and sucrose metabolism, and glyoxylate and dicarboxylate metabolism (Figure 5b). Enrichment was also observed in the brassinosteroid biosynthesis and autophagy (other types) pathways (Table S4).

To dissect the cause of the deflated papilla cell phenotype in the *sd* mutant, we analyzed genes involved in morphogenesis and polar growth of papilla cells, brassinosteroid (BR) biosynthesis genes regulating cell elongation and turgor pressure [36,37,38], auxin pathway genes essential for stigma development, sugar transport and sugar signaling genes, and genes related to cell turgor and vacuole development (Table S5). The results showed that the core transcription factor *BZR1* in the BR signaling pathway was significantly downregulated; the sugar transporters *SWEET11* (with log_2_FC ranging from 0.2113 to 1.5409) and *SWEET12* (with log_2_FC ranging from 0.1183 to 1.1775) were significantly upregulated, while *SWEET15* (with log_2_FC ranging from −1.1646 to −0.0241) was significantly downregulated (Figure 5c); auxin-related genes exhibited irregular expression fluctuations (some up-, some downregulated); no significant differences were observed in the remaining genes. These findings suggest that reduced cell elongation capacity, insufficient turgor maintenance, and disturbed sugar transport may be the main causes of the deflated papilla cell phenotype in the *sd* mutant.

To elucidate the mechanism underlying random pollen tube growth, we analyzed genes related to stigma secretory functions (e.g., lipid transfer proteins, stigma-specific adhesion proteins, and secretion pathway proteins), cell wall remodeling genes essential for pollen tube penetration (e.g., cutin and wax synthesis genes, cell wall modification enzymes, and callose synthases), and as self-incompatibility-related genes [39] (Table S5). The results showed no significant differences in the expression of the above genes, indicating that the random pollen tube growth on the *sd* mutant stigma is not caused by self incompatibility but rather results from structural defects in stigma development. Meanwhile, most genes involved in normal development and secretion showed no significant differences, suggesting that the *sd* mutant stigma is structurally developed and not congenitally malformed; instead, it loses function at later stages, preventing mature pollen grains from inducing pollen tube penetration.

We further analyzed genes associated with cellular senescence and programmed cell death (PCD) [40,41,42] (Table S5). The results revealed that the pro-senescence transcription factors *ANAC029* (*VAP*), *ANAC083* (*VNI2*), and *ANAC030* (*VND7*), the autophagy protein ATG4B, and the PCD related genes *MC1*, *MC9*, and *XCP1/2* were all significantly upregulated (Figure 5c), suggesting that the reduced seed set in the *sd* mutant is closely associated with premature senescence and loss of viability of papilla cells.

We also analyzed the expression of osmotic regulation related genes and found that calmodulins, calcium dependent protein kinases, and potassium transporters exhibited varying degrees of significant change (Table S6). This indicates a collapse in calcium homeostasis during papilla cell senescence and PCD. Furthermore, analysis of hormone pathway related genes showed that mutation of the *CRC* gene led to significant changes in five hormone pathway related genes (Table S7), suggesting that *CRC* may be involved in a complex hormonal regulatory network. This is consistent with the results of the cis-acting element analysis of *CRC*.

### 2.8. qRT-PCR Validation of Transcriptome Data

To verify the reliability of the transcriptome sequencing results, ten differentially expressed genes in the stigmas of *sd* mutant versus wild-type plants were randomly selected, namely *BZR1*, *SWEET12*, *NAC029*, *NAC083*, *MC1*, *CAT2*, *APX6*, *FSD1*, *SWEET11* and *SWEET15*. Their expression levels were examined by qRT-PCR. The results showed that the expression patterns of these genes in the *sd* mutant were fully consistent with the transcriptome data (Figure S6), confirming the accuracy and reliability of the transcriptome analysis in this study.

## 3. Discussion

### 3.1. Evolutionary Conservation and Subfamily Differentiation of the YABBY Gene Family in Brassicaceae

In this study, four representative species of Brassicaceae were selected for systematic analysis of the YABBY gene family, offering unique comparative advantages in species design. As a paraphyletic ancestor of *Brassica* species, *Arabidopsis* provides the most comprehensive genomic information. *Brassica rapa* (*AA*) and *Brassica oleracea* (*CC*) are the two diploid progenitor species of *Brassica napus* (*AACC*) [43], which establishes an ideal framework for tracing the expansion, retention, and loss of YABBY genes following allopolyploidization. The *YABBY* family is divided into five subfamilies in most angiosperms (*FIL/YAB3*, *YAB2*, *YAB5*, *INO*, *CRC*), among which *FIL* and *YAB3* are grouped into the same subfamily due to high sequence similarity and an ancient gene duplication event [17]. Phylogenetic analysis conducted in this study across four Brassicaceae species clearly classified *YABBY* genes into six subfamilies, with *YAB1* and *YAB3* forming distinct clades, indicating stable functional differentiation between them in Brassicaceae. This divergence likely reflects the evolutionary specialization in Brassicaceae for leaf polarity establishment (*YAB1/FIL*) versus floral organ development (*YAB3*). Previous studies have shown that *CRC* shares a common ancestor with *YAB1*, while *INO* is a sister gene to the *CRC/YAB1* ancestor [10]. Based on this evolutionary framework, the separation of *YAB1* and *YAB3* in Brassicaceae, together with the clustering of *YAB2* and *YAB5* (Figure 1), may suggest that after at least four rounds of duplication events in the ancestral *YABBY* genes of angiosperms, different lineages experienced differential retention and functional divergence in Brassicaceae [18]. Furthermore, the distribution of *YABBY* genes on the *A* and *C* subgenomes of *B. napus* in this study largely corresponds to that in *B. rapa* and *B. oleracea*, with only one exceptional copy on chromosome A09 (Figure 2), indicating that after allopolyploidization, *YABBY* genes have been primarily directly inherited from the progenitors rather than undergoing extensive new rounds of expansion or loss. This finding is consistent with the observation by Xia et al. [17] in *B. napus* that extensive duplication and loss occurred after polyploidization, while the overall gene number remained largely similar to that of the progenitor species.

### 3.2. Structural Conservation and Interspecific Functional Divergence of the CRC Subfamily

*CRC* is the only member of the YABBY family that specifically regulates reproductive organ development (particularly carpels and nectaries) in angiosperms [1]. In this study, we found that the CRC proteins from four representative Brassicaceae species are nearly identical in terms of amino acid length (179–181 aa), molecular weight (approximately 19.6–19.8 kDa), isoelectric point (pI 9.56–9.66), nuclear localization (Table 1 and Table S1), conserved motif composition (four motifs), and gene structure (7 exons/6 introns) (Figure 3), indicating that the biochemical function and basic regulatory unit of *CRC* have undergone strong purifying selection in Brassicaceae. Notably, *CRC* in Arabidopsis and *B. napus* contains both 5′UTR and 3′UTR, whereas *CRC* in *B. rapa* and *B. oleracea* lacks detectable UTRs (Figure 3). This may be due to the completeness of genome annotations, or it might suggest that UTRs were regained or retained during allopolyploidization.

All *CRC* promoters are enriched in light-responsive, MeJA-responsive, and promoter/enhancer-related cis-elements (Table S2; Figure S5), representing a core module of *CRC* transcriptional regulation. However, the *Arabidopsis CRC* promoter uniquely contains a circadian-related element. In contrast, those of *B. rapa*, *B. oleracea*, and the two *CRC* copies in *B. napus* additionally contain auxin-responsive elements (2–5 copies), defense and stress-responsive elements (1 copy each), and an endosperm expression element (1 copy each). Notably, the number of auxin-responsive elements in the two *CRC* copies of *B. napus* (4 copies) is higher than that in *B. rapa* (2 copies) but lower than that in *B. oleracea* (5 copies). This likely reflects a rebalancing of the cis-regulatory network following allopolyploidization. These species-specific cis-elements may confer expression plasticity to *CRC* in hormone responses (particularly auxin) [44,45], circadian rhythms, and stress responses, thereby influencing its functional output across different species.

### 3.3. Functional Inference of CRC in B. napus

It has been reported that pollen attachment to the stigma induces Ca^2+^ accumulation, leading to stigma papilla wilting [46]. In the *sd* mutant (carrying a double deletion of *BnYAB6.1* and *BnYAB6.2*), the flower buds exhibit stigma papilla wilting even before anthesis [34]. Transcriptomic analysis of stigmas from 2 to 4 mm buds revealed significant changes in the expression of calmodulin and calcium-dependent protein kinases, among others, to varying degrees (Table S6). However, the causal relationship between these changes remains unknown. *CRC* not only plays an essential role in carpel development [1] but also functions in floral meristem termination, often manifesting as increased floral organ numbers or altered sex determination [27,47,48]. In the *sd* mutant, however, no defects such as unfused carpel apices or abnormalities in floral meristem termination (e.g., increased organ numbers) were observed, and the expression levels of floral meristem termination-related genes (e.g., *WUS*, *AG*) showed no significant changes (Table S5). These results suggest that in *B. napus*, *CRC* may have lost some of the functions retained in *Arabidopsis* (carpel apical closure and floral meristem termination), while retaining and enhancing the function affecting stigma maturation and papilla cell viability. Future ectopic expression experiments (e.g., transforming the *B. napus CRC* gene into the *Arabidopsis* crc mutant) combined with comparative ChIP-seq analyses will help clarify the molecular mechanisms underlying the functional divergence of *CRC*.

### 3.4. Analysis of the Triggering Mechanism of PCD in Stigma Papilla Cells of the Rapeseed sd Mutant

**Figure 6:**
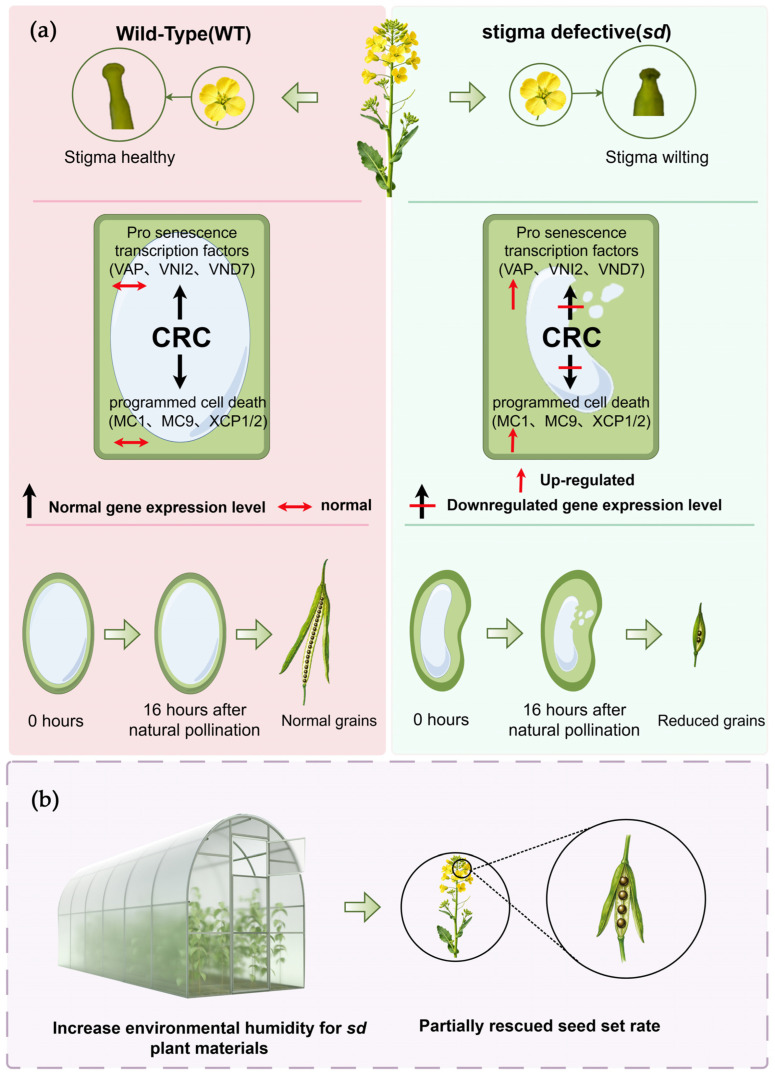
The molecular mechanism underlying the control of carpel development by *CRC*. (**a**) The molecular mechanism underlying the control of carpel development by *CRC* in wild-type and *sd* mutants; (**b**) the effect of increased ambient humidity on the seed setting rate of the *sd* mutant.

In the *sd* mutant, although pro-senescence transcription factors (*ANAC029/VAP*, *ANAC083/VNI2*, *ANAC030/VND7*), the autophagy protein ATG4B, and PCD effector genes (*MC1*, *MC9*, *XCP1/2*) [40,41,42,49] were all significantly upregulated. However, classical senescence-associated genes (*WRKY*, *SAG*, *BI-1*, *DAD1*) and genes involved in ABA and ethylene signaling pathways showed no significant changes [50]. This indicates that senescence in the papilla cells of the *sd* mutant does not occur through typical hormone-induced senescence pathways (e.g., ABA/ethylene-dependent senescence), but may instead proceed via a non-classical pathway. One possibility is a “stress-responsive” PCD triggered directly by loss of cell wall integrity, osmotic stress, or collapse of calcium homeostasis [51]. When *CRC* is absent, papilla cells may remain in a persistently low-turgor state, and this prolonged cellular stress could bypass classical senescence signals and directly activate a PCD program mediated by specific NAC transcription factors (e.g., VNDs; Figure 6a). This hypothesis is consistent with the observed timing of vacuole rupture (16 h after natural pollination): during their attempt to support pollen recognition and penetration, the combination of mechanical pressure and intrinsic defects leads to vacuolar membrane rupture and the initiation of terminal PCD.

Interestingly, previous observations showed that the using of selfing bags or double-layer netting (which increases ambient humidity) significantly improved seed set in the *sd* mutant (Figure 6b and Figure S7) [34]. We speculate that high humidity conditions may temporarily alleviate the decline in papilla cell turgor by reducing water evaporation from the stigma surface [52,53,54], thereby delaying cell collapse and PCD initiation. Alternatively, high humidity may affect stigma surface secretions and reactive oxygen species homeostasis [55], thereby suppressing premature PCD activation. This phenomenon is likely consistent with the significant differences in the expression of sugar transport- and calcium signaling-related genes observed in the *sd* mutant in this study. This suggests that high humidity may partially compensate for deficiencies in cellular osmotic regulation. While we have not directly examined the expression of PCD-related genes in the stigmas of the *sd* mutant under high-humidity conditions, this observation provides an important clue for future investigations into the causal chain linking humidity, PCD, and seed set. Furthermore, this finding suggests that in rapeseed breeding, moderate increases in field humidity during flowering (e.g., through rational irrigation or the use of glasshouses) could help mitigate yield losses caused by *CRC* functional defects.

## 4. Materials and Methods

### 4.1. Plant Materials and Growth Conditions

The materials used for transcriptome sequencing were derived from a high-generation segregating population of the rapeseed *sd* mutant (Wuhan, China). The *sd* mutant is a stably inherited mutant line characterized and published in our previous study [27]. From this population, individuals exhibiting extreme phenotypes—either wild-type or mutant stigmas—were selected. All plants were cultivated in a breeding greenhouse under controlled environmental conditions, with a constant temperature of 22 °C and a 16 h light/8 h dark photoperiod. Regular watering and fertilization were applied to ensure optimal plant development. For sample collection, stigmas were excised from flower buds measuring 2–4 mm in length using sterile surgical blades. The harvested tissues were immediately placed into sterile microcentrifuge tubes, snap-frozen in liquid nitrogen, and subsequently stored at −80 °C for downstream transcriptome sequencing and gene expression validation. For each phenotype, three biological replicates were prepared, with each replicate consisting of pooled stigmas from 30 individual flower buds to ensure the representativeness and reproducibility of the samples.

### 4.2. Identification of the YABBY Gene Family in Brassicaceae Species

The protein sequences of *A. thaliana* YABBY (YAB) were retrieved from The Arabidopsis Information Resource (TAIR) database (https://www.arabidopsis.org/, accessed on 12 March 2026). Homologous YAB protein sequences from *B. rapa* (genome version Brara_Z1_V1), *B. oleracea* (Braol_HDEM_V1.0), and *B. napus* (Brana_ZS_HZAU_V1.0) were retrieved from the Brassicaceae Database (BRAD, http://brassicadb.cn/, accessed on 12 March 2026). To identify YAB family members across the four species, BLASTP searches were performed using *Arabidopsis* YAB sequences as queries, with an E-value threshold of E-value < 1 × 10^−10^ set for homologous sequence filtering. The physicochemical properties of the identified YAB proteins, including the isoelectric point (pI), molecular weight (MW), and amino acid length, were analyzed using the ExPASy ProtParam online tool (http://web.expasy.org/proparam/, accessed on 2 April 2026). Furthermore, the subcellular localization of these YAB proteins was predicted using the DeepLoc-2.0 server (http://services.healthtech.dtu.dk/services/DeepLoc-2.0/, accessed on 20 April 2026).

### 4.3. Bioinformatic Analysis of the YABBY Gene Family

The chromosomal distribution of YAB genes was determined by extracting location data from the GFF3 annotation files of the four *Brassicaceae* species. These results were subsequently visualized using TBtools-II software (v2.441). Multiple sequence alignment of the YAB proteins from the four Brassicaceae species was performed using MEGA12 software (v12.1.2) with default parameters. Based on the alignment results, a phylogenetic tree was constructed using the Maximum Likelihood (ML) method. To evaluate the reliability of the phylogenetic branches, bootstrap analysis was conducted with 1000 replicates. The phylogenetic tree was visualized using the TVBOT online tool (https://www.chiplot.online/tvbot.html, accessed on 16 April 2026). Synteny relationships of the YAB gene family among the four species were analyzed using the MCscanX toolkit integrated within TBtools-II, and the synteny map was generated via the Multiple Synteny Plot plugin. The conserved motifs of YAB proteins were identified using the MEME Suite online server (https://meme-suite.org/meme/, accessed on 15 March 2026), with the maximum number of motifs set to 10 and all other parameters maintained at default settings. Additionally, the conserved domains of the YAB proteins were retrieved and analyzed via the NCBI Conserved Domain Database (CDD) (https://www.ncbi.nlm.nih.gov/cdd, accessed on 15 March 2026). The cis-acting elements in the promoter regions of YAB genes were predicted using the PlantCARE online tool (https://bioinformatics.psb.ugent.be/webtools/plantcare/html/, accessed on 13 March 2026). All aforementioned analytical results were visualized using TBtools-II software. Tissue-specific expression data for *B. napus* YAB genes were retrieved from the BnIR database (https://yanglab.hzau.edu.cn/, accessed on 23 April 2026), and expression heatmaps were generated using GraphPad Prism software (v8.0.1(244)).

### 4.4. Transcriptome Analysis

The collected stigma samples (with three independent biological replicates for each sample) were submitted to Shanghai Personal Biotechnology Co., Ltd. (Shanghai, China) for cDNA library construction. Sequencing was performed on the Illumina platform using a paired-end strategy (2 × 150 bp). Raw data were processed using Cutadapt (v 1.15) to remove adapter sequences, and low-quality reads with an average quality score of <Q20 were filtered out to obtain clean reads. Clean reads were mapped to the *B. napus* reference genome (ZS11) using HISAT2. Gene read counts were quantified with HTSeq, and Fragments Per Kilobase of transcript per Million mapped reads (FPKM) was employed as the metric for gene expression levels. Differential expression analysis was performed using DESeq (v1.20.0), with the filtering thresholds for differentially expressed genes (DEGs) set at |log_2_FoldChange| > 1 and *P*-adj < 0.05. The DEGs were subjected to Gene Ontology (GO) functional enrichment analysis using topGO, while Kyoto Encyclopedia of Genes and Genomes (KEGG) pathway enrichment analysis was performed using KOBAS. A threshold of *P*-adj < 0.05 was applied to identify significantly enriched terms and pathways.

### 4.5. Validation of Gene Expression

The qRT-PCR assays were performed using a CFX96 Touch Real-Time PCR Detection System (Bio-Rad, Hercules, CA, USA), following the experimental procedure previously described by [8] The relative mRNA expression levels were calculated using the 2^−∆∆CT^ method as previously described by Livak and Schmittgen [56]. All reactions were performed in triplicate to ensure technical and biological reproducibility. All statistical analyses were performed using GraphPad Prism software (v8.0.1(244)). Significant differences were determined via one-way analysis of variance, with statistical significance defined as *p* < 0.05 and extreme significance as *p* < 0.01.

## 5. Conclusions

In this study, the YABBY gene family was systematically identified and characterized in four representative Brassicaceae species (*B. napus*, *B. rapa*, *B. oleracea*, and *A. thaliana*). A total of 50 YABBY genes were identified and classified into six subfamilies (YAB1, YAB2, YAB3, INO, YAB5, and CRC). The CRC subfamily is structurally highly conserved in Brassicaceae but has undergone interspecific functional divergence. Unlike in *Arabidopsis*, the rapeseed *CRC* lost the functions of regulating carpel tip closure and floral meristem termination, while retaining core functions in nectary development, stigma morphogenesis, and papilla cell viability. Interspecific differences in cis-acting elements (e.g., enrichment of auxin-responsive elements in rapeseed) may represent the molecular basis for this functional divergence. The reduced seed set in the *sd* mutant (double deletion of *BnYAB6.1* and *BnYAB6.2*) can be attributed to a cascade of morphological defects, signaling dysregulation, and stress-induced programmed cell death (PCD): *CRC* deletion leads to downregulation of BR signaling and dysregulation of sugar transport, resulting in a loss of papilla cell turgor and surface collapse, which creates a physical barrier to pollen tube penetration; the auxin pathway genes show irregular expression, indicating a complex feedback network between *CRC* and auxin signaling; chronic low turgor, calcium homeostasis collapse, and abnormal sugar metabolism activate a non-canonical senescence pathway (upregulation of *VAP/VNI2/VND7* and *MC1/MC9/XCP1/2*, with no changes in the ABA/ethylene pathway), ultimately leading to vacuole rupture in papilla cells and permanent loss of stigma function. So, it may be proposed that increased ambient humidity can partially rescue the seed set of the *sd* mutant by delaying PCD, providing a theoretical basis for humidity management during rapeseed flowering. This study systematically elucidates the evolutionary characteristics of the YABBY gene family in Brassicaceae and, for the first time in rapeseed, reveals the multi-layer molecular mechanism by which *CRC* regulates stigma development and seed set, thereby providing important genetic resources and theoretical support for the genetic improvement of yield traits in rapeseed.

## Figures and Tables

**Table 1:** Physicochemical properties and subcellular localization prediction of YABBY genes in Brassicaceae.

No.	Gene ID	Gene Name	Chromosome	Protein Length (aa)	MW (Da)	pI	Subcellular Localization Predicted
1	BnaA03T0219600ZS	BnYAB1.1	A03	225	25,087.64	7.7	Nucleus
2	BnaC03T0258500ZS	BnYAB1.2	C03	226	25,244.81	7.7	Nucleus
3	BnaA04T0285400ZS	BnYAB1.3	A04	233	26,094.82	6.44	Nucleus
4	BnaC04T0603400ZS	BnYAB1.4	C04	233	26,067.8	6.44	Nucleus
5	BnaA07T0201600ZS	BnYAB1.5	A07	218	24,667.23	7.11	Nucleus
6	BnaC06T0210100ZS	BnYAB1.6	C06	228	25,592.39	7.68	Nucleus
7	BnaA06T0050400ZS	BnYAB2.1	A06	171	19,276.73	9.75	Nucleus
8	BnaC05T0063000ZS	BnYAB2.2	C05	188	21,232.05	9.54	Nucleus
9	BnaA08T0299400ZS	BnYAB2.3	A08	188	21,205.04	9.51	Nucleus
10	BnaC08T0183800ZS	BnYAB2.4	C08	189	21,259.11	9.51	Nucleus
11	BnaA09T0665900ZS	BnYAB2.5	A09	188	21,005.86	9.68	Nucleus
12	BnaC08T0530700ZS	BnYAB2.6	C08	188	21,007.83	9.57	Nucleus
13	BnaA09T0000600ZS	BnYAB3.1	A09	238	26,210.85	8.66	Nucleus
14	BnaA09T0010100ZS	BnYAB3.2	A09	238	26,210.85	8.66	Nucleus
15	BnaA07T0117200ZS	BnYAB4.1	A07	224	25,325.51	5.78	Nucleus
16	BnaC07T0173800ZS	BnYAB4.2	C07	235	26,454.82	5.41	Nucleus
17	BnaA09T0452100ZS	BnYAB4.3	A09	233	26,130.44	5.91	Nucleus
18	BnaC05T0206300ZS	BnYAB4.4	C05	226	25,131.23	5.51	Nucleus
19	BnaA03T0236900ZS	BnYAB5.1	A03	164	18,311.95	9.47	Nucleus
20	BnaC03T0279900ZS	BnYAB5.2	C03	117	12,950.84	9.45	Nucleus
21	BnaA07T0307300ZS	BnYAB6.1	A07	179	19,617.38	9.66	Nucleus
22	BnaC06T0356800ZS	BnYAB6.2	C06	179	19,620.38	9.56	Nucleus
23	BraA03t11711Z	BrYAB1.1	A03	225	25,087.64	7.7	Nucleus
24	BraA04t18831Z	BrYAB1.2	A04	286	32,659.43	9.05	Nucleus
25	BraA07t30030Z	BrYAB1.3	A07	234	26,611.59	8.15	Nucleus
26	BraA06t23754Z	BrYAB2.1	A06	190	21,387.21	9.54	Nucleus
27	BraA08t35422Z	BrYAB2.2	A08	187	21,103.93	9.51	Nucleus
28	BraA09t41932Z	BrYAB2.3	A09	188	21,005.86	9.68	Nucleus
29	BraA09t35690Z	BrYAB3	A09	238	26,210.85	8.66	Nucleus
30	BraA07t29143Z	BrYAB4.1	A07	263	29,795.88	6.21	Nucleus
31	BraA09t39616Z	BrYAB4.2	A09	233	26,093.37	5.82	Nucleus
32	BraA03t11900Z	BrYAB5	A03	164	18,311.95	9.47	Nucleus
33	BraA07t31141Z	BrYAB6	A07	180	19,745.52	9.66	Nucleus
34	BolC3t15340H	BoYAB1.1	C03	294	33,137.72	9.27	Nucleus
35	BolC4t28650H	BoYAB1.2	C04	278	31,431.01	8.82	Nucleus
36	BolC6t37651H	BoYAB1.3	C06	294	27,608.76	8.5	Nucleus
37	BolC5t29417H	BoYAB2.1	C05	188	21,232.05	9.54	Nucleus
38	BolC8t48831H	BoYAB2.2	C08	189	21,245.08	9.51	Nucleus
39	BolC8t52528H	BoYAB2.3	C08	188	20,984.79	9.57	Nucleus
40	BolC9t52950H	BoYAB3	C09	238	26,081.65	8.33	Nucleus
41	BolC7t42568H	BoYAB4.1	C07	232	26,116.44	5.39	Nucleus
42	BolC5t30970H	BoYAB4.2	C05	260	29,217.94	6.32	Nucleus
43	BolC3t15566H	BoYAB5	C03	164	18,249.81	9.32	Nucleus
44	BolC6t39360H	BoYAB6	C06	180	19,748.52	9.56	Nucleus
45	AT2G45190	AtYAB1	Chr2	229	25,779.42	6.78	Nucleus
46	AT1G08465	AtYAB2	Chr1	184	20,700.52	9.40	Nucleus
47	AT4G00180	AtYAB3	Chr4	240	26,337.96	8.67	Nucleus
48	AT1G23420	AtINO	Chr1	262	29,632.54	6.42	Nucleus
49	AT2G26580	AtYAB5	Chr2	164	18,505.19	9.47	Nucleus
50	AT1G69180	AtCRC	Chr1	181	19,722.48	9.56	Nucleus

Note: aa: amino acid; MV (Da): Molecular Weight (Dalton); pI: Isoelectric Point; A and C indicate the A and C subgenomes of *Brassica napus*, respectively; Chr: Chromosome.

## Data Availability

The raw RNA-seq data generated and analyzed in this study have been deposited in the NCBI Sequence Read Archive (SRA) database under BioProject accession number PRJNA1479095. All data generated or analyzed during this study are included in this published article and its Supplementary Materials.

## Supplementary Materials

The following supporting information can be downloaded at: https://www.mdpi.com/article/10.3390/ijms27135740/s1.
