# Supplementary material for: The Phylogeny of Brassicaceae YABBYs and the CRC-Mediated Regulation of Stigma Development in Brassica napus
Source: Int J Mol Sci. 2026 Jun 25;27(13):5740. doi: 10.3390/ijms27135740 (PMC13361579; doi:10.3390/ijms27135740)
Supplement: Supplementary file 1 [file ijms-27-05740-s001.zip › Supplementary Figure.pdf]

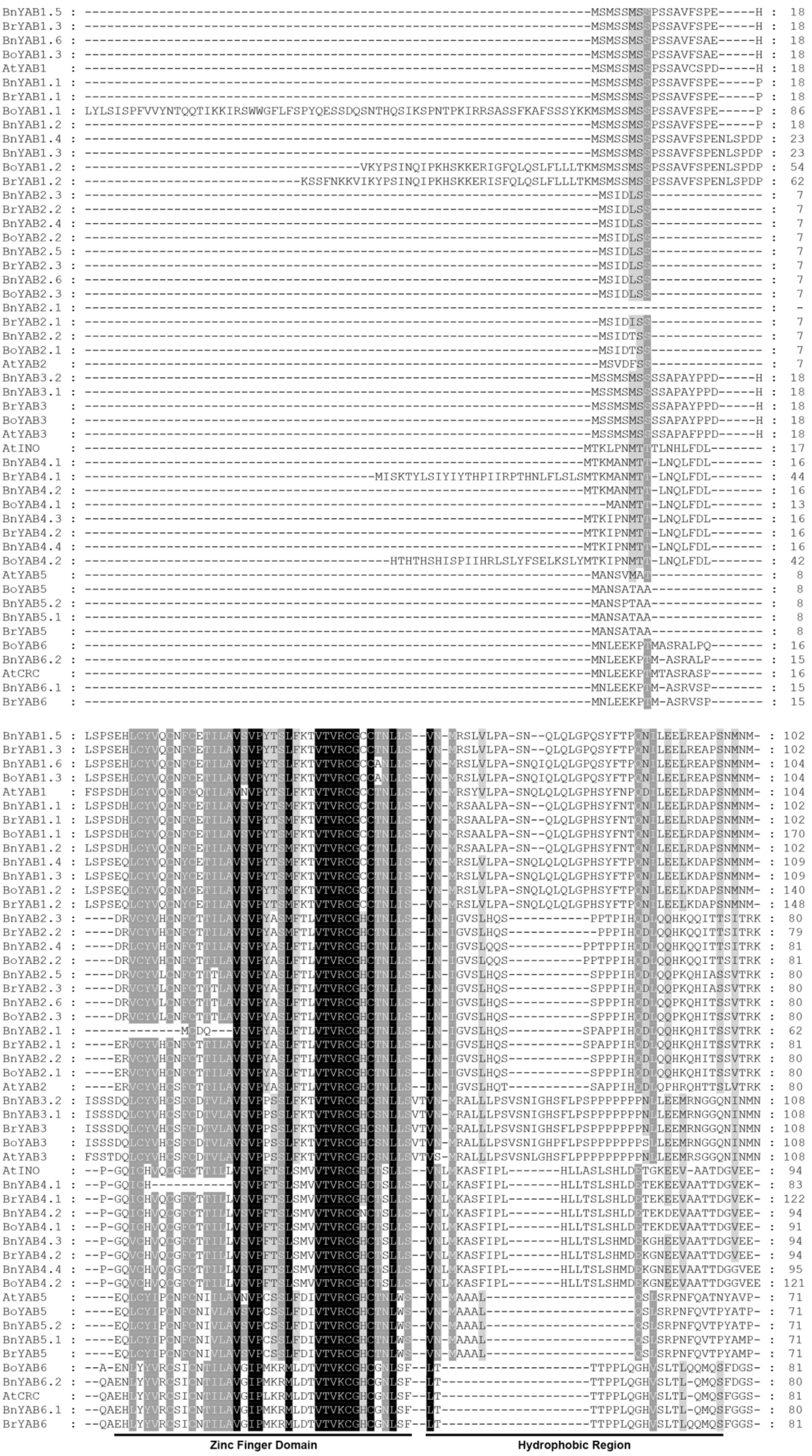
Zinc Finger Domain
Hydrophobic Region

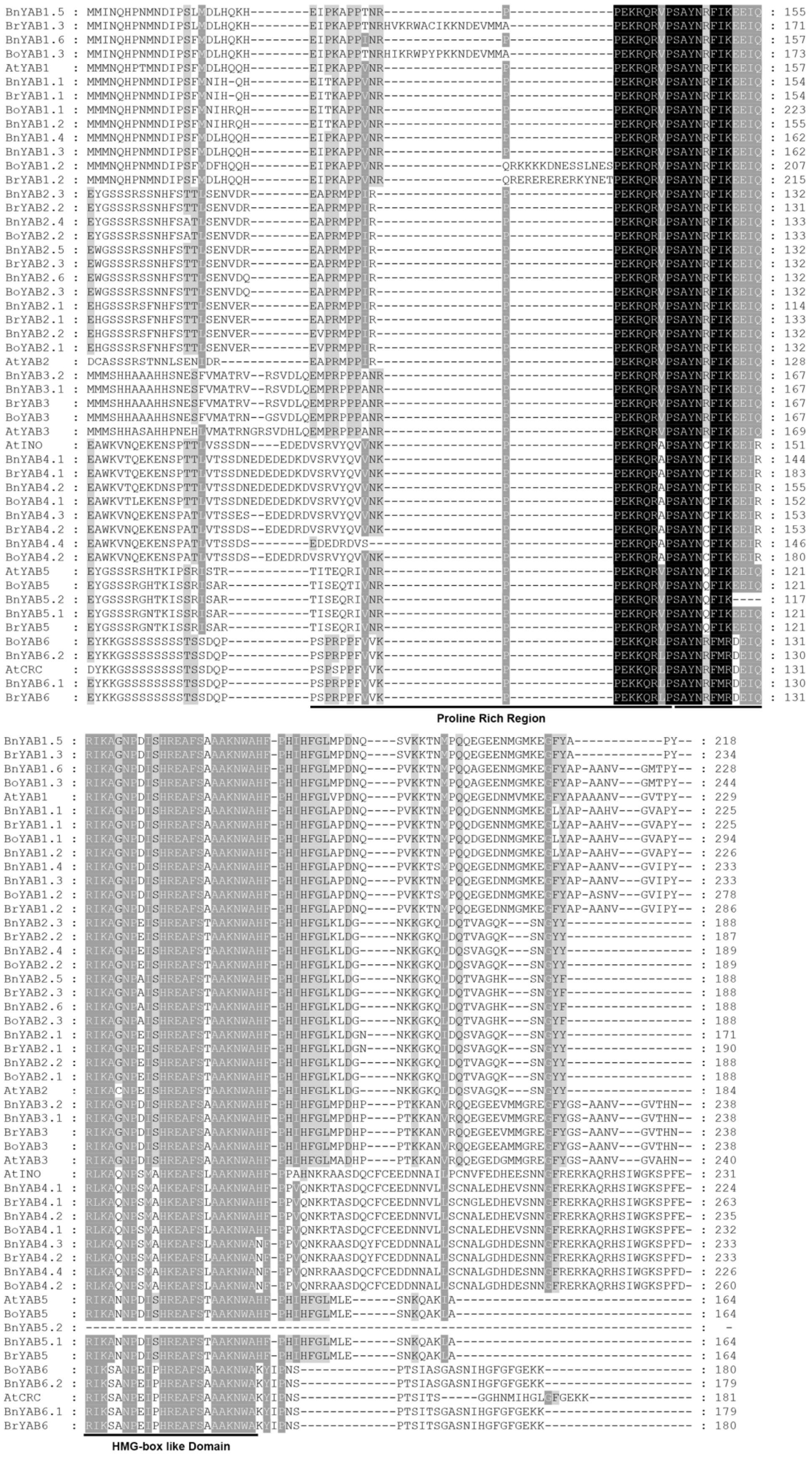

Figure S1 Alignment of YABBY proteins from four Brassicaceae species

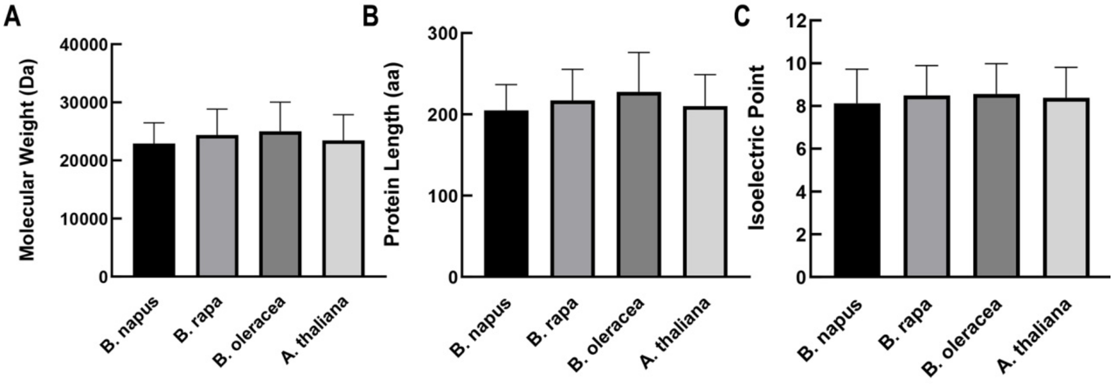

Figure S2 Statistics of molecular weight, amino acid number, and isoelectric point of YABBY proteins from four Brassicaceae species

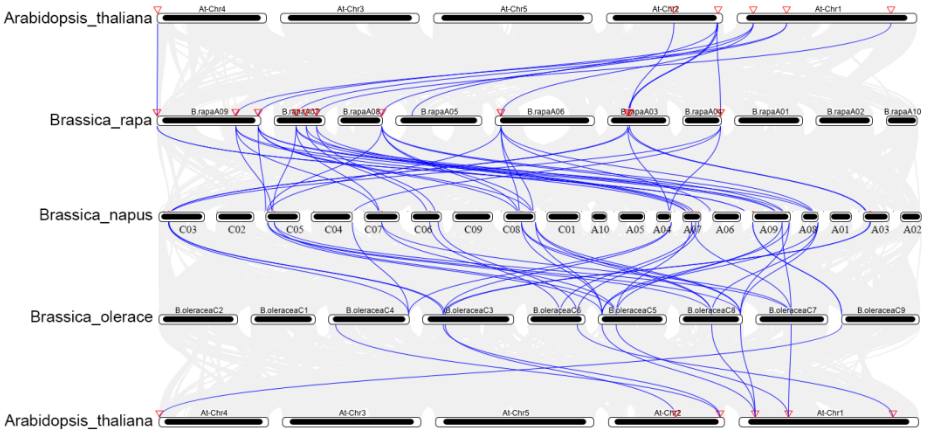

Figure S3 Synteny analysis of YABBY genes in four representative Brassicaceae species

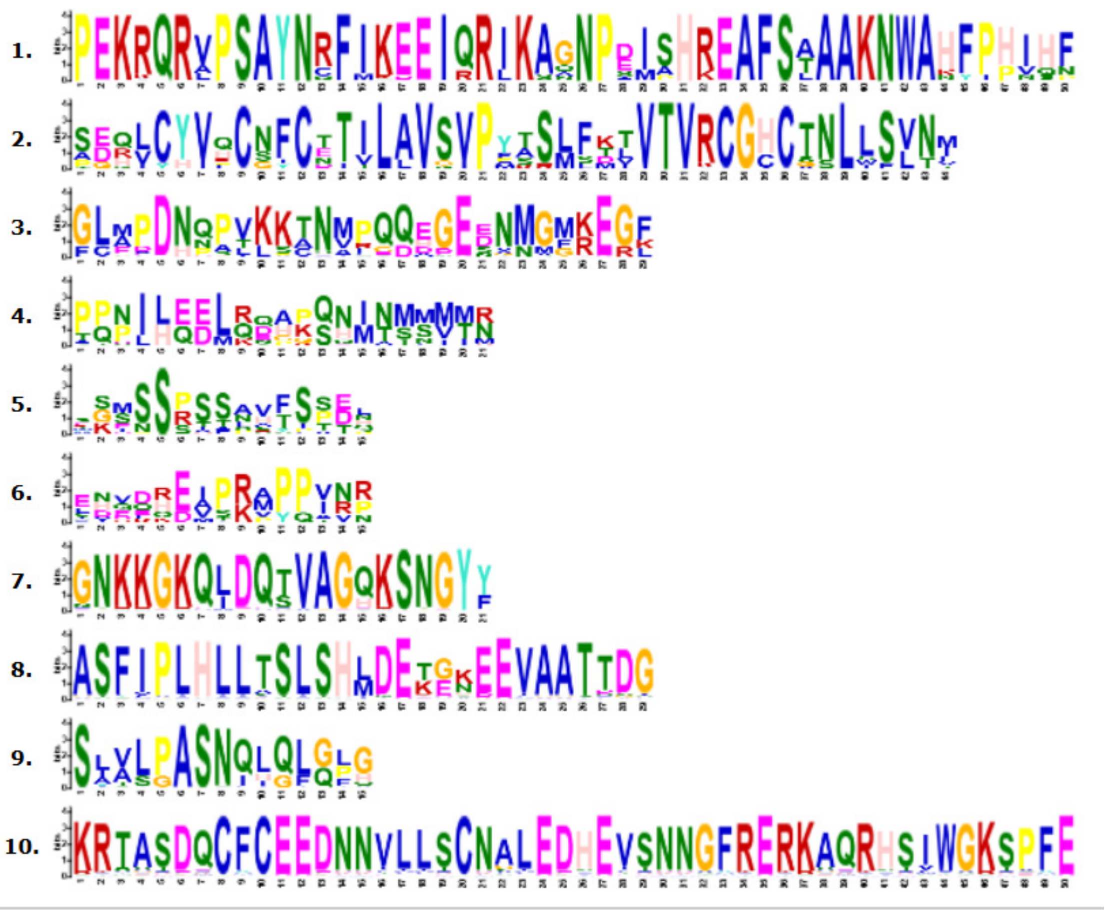

Figure S4 Sequence logos of the 10 conserved motifs predicted by MEME

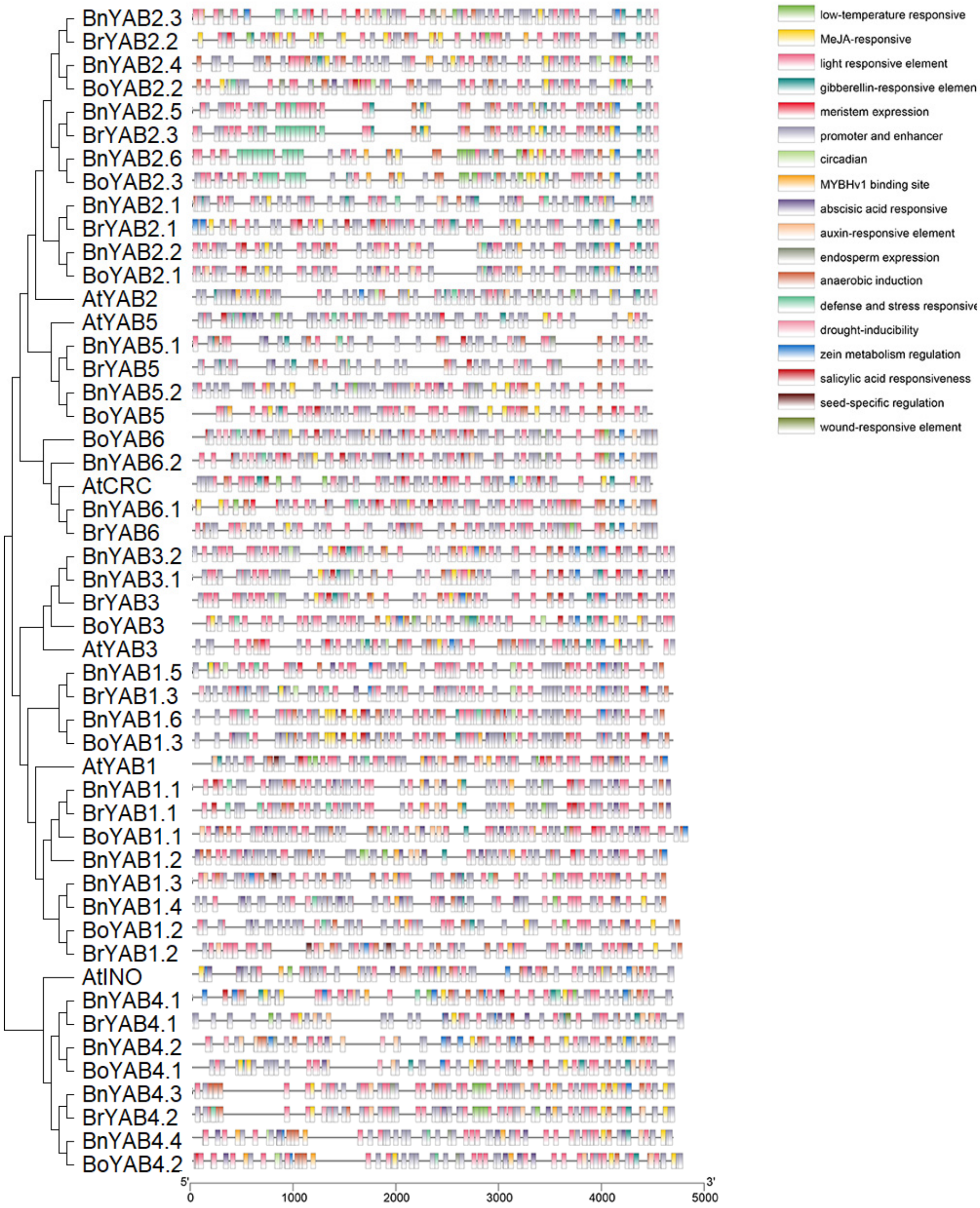

Figure S5 Cis-acting element analysis of YABBY genes in four representative Brassicaceae species

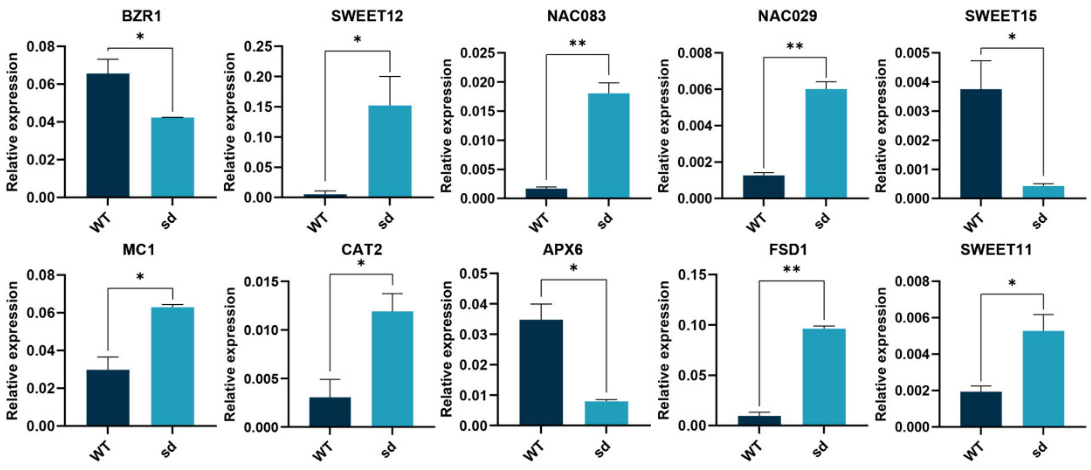

Figure S6 Validation of expression levels of selected differentially expressed genes by qRT-PCR

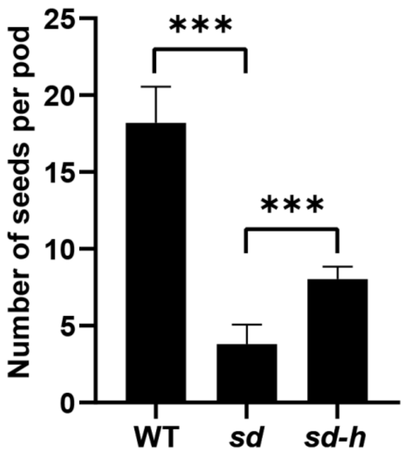

Figure S7 Number of seeds per pod in wild-type, *sd* and *sd-h*

Values represent means ± SD (n ≥ 3). Bars with different letters are significantly different ($P < 0.05$, one-way ANOVA). WT, wild type; *sd*, *sd* mutant; *sd-h*, high humidity-treated *sd* mutant.
